# Birth Spacing of Pregnant Women in Nepal: A Community-Based Study

**DOI:** 10.3389/fpubh.2016.00205

**Published:** 2016-09-19

**Authors:** Rajendra Karkee, Andy H. Lee

**Affiliations:** ^1^BP Koirala Institute of Health Sciences, School of Public Health and Community Medicine, Dharan, Nepal; ^2^School of Public Health, Curtin University, Perth, WA, Australia

**Keywords:** fertility control, birth interval, birth spacing, Nepal

## Abstract

**Background:**

Optimal birth spacing has health advantages for both mother and child. In developing countries, shorter birth intervals are common and associated with social, cultural, and economic factors, as well as a lack of family planning. This study investigated the first birth interval after marriage and preceding interbirth interval in Nepal.

**Methods:**

A community-based prospective cohort study was conducted in the Kaski district of Nepal. Information on birth spacing, demographic, and obstetric characteristics was obtained from 701 pregnant women using a structured questionnaire. Logistic regression analyses were performed to ascertain factors associated with short birth spacing.

**Results:**

About 39% of primiparous women gave their first child birth within 1 year of marriage and 23% of multiparous women had short preceding interbirth intervals (<24 months). The average birth spacing among the multiparous group was 44.9 (SD 21.8) months. Overall, short birth spacing appeared to be inversely associated with advancing maternal age. For the multiparous group, Janajati and lower caste women, and those whose newborn was female, were more likely to have short birth spacing.

**Conclusion:**

The preceding interbirth interval was relatively long in the Kaski district of Nepal and tended to be associated with maternal age, caste, and sex of newborn infant. Optimal birth spacing programs should target Janajati and lower caste women, along with promotion of gender equality in society.

## Introduction

Birth spacing is an essential component of family planning and fertility control (1). Optimal spacing between successive births or pregnancies has health advantages for both mother and child. Either short (<18 months) or long (>59 months) birth intervals can increase the risk of adverse perinatal and maternal outcomes (2). Babies born either at short or long birth intervals are likely to be preterm, small for gestational age, and have low birthweight, while their mothers may suffer maternal anemia, fetal loss, premature rupture of membranes, and eclampsia (3–6). Therefore, effective birth spacing is important not only for population control but also for improving maternal and child health.

In developing countries, short birth intervals are prevalent and mostly unintended (7). Nepal is predominantly a patriarchal country with diversified cultural and religious practices. Limited qualitative works suggests gender disparity is high, and women often do not have full control of their own reproductive life (8). Indeed, the median age at marriage increased by only 1 year during the past decade from 16.6 years in 2001 to 17.5 years in 2011 (9, 10). The early marriage is usually followed by the first child birth without much delay. The median maternal age at first birth is 20.2 years. Subsequent births are likely, especially if the first child is not a boy in this son-preferred society with expectation to be looked after at the old age (11). Such societal and cultural values contribute to high fertility with short birth intervals in the past and still persist as a challenge for family planning. The fertility rate in Nepal was above 5 until the early 1990s, above 4 in 2001, before dropping to 2.6 in 2011 (9, 10). Two to three children remain common among Nepalese couples.

Besides societal and cultural norms, various factors also contribute to high fertility and short birth spacing at individual, household, and community levels. These include illiteracy, early marriage, lack of family planning methods, and poverty (11). Knowledge on birth spacing and the underlying characteristics is required to understand reproductive patterns and fertility behavior, with the ultimate goal to develop effective family planning strategies. This study aimed to investigate the first birth interval after marriage and preceding interbirth interval and their characteristics for women living in central Nepal.

## Materials and Methods

### Setting

This community-based study was undertaken in Kaski district, located in the relatively developed central hills region of Nepal. The district has a central valley popularly known as Pokhara city. The rural areas stretch out of the valley in hilly terraces. Kaski has 75% female literacy, approximately 134,100 married women with 13,800 expected pregnancies annually (12).

### Study Design and Participants

The results were derived from a large prospective cohort study of delivery service utilization and pregnancy outcomes between December 2011 and November 2012. Details concerning the study design and sampling had been described elsewhere (13, 14). Briefly, five urban wards and seven rural areas within the Kaski district were randomly selected to recruit participants’ representative of the target population. The final sample is composed of 701 pregnant women of 5 months or more gestational age who were followed up until 6 months postpartum.

### Data Collection and Ethics

The questionnaire used was adapted from the validated Nepal Demographic and Health Survey. Its validity and reliability had been tested on 50 pregnant women for cultural appropriateness and understanding. Fifteen female data enumerators recruited the pregnant women in their local community and conducted face-to-face interviews at the home of participants to collect information on socio-demographic characteristics, obstetric, and family planning knowledge. Maternal age at recruitment, age at marriage, and the age of their youngest child (for multiparous women) were also recorded.

The study protocol was approved by the Human Research Ethics Committee of Curtin University (approval number HR 130/2011), Ethical Review Board of the Nepal Health Research Council (approval number 88/2011), and the District Public Health Office of Kaski. An information sheet was distributed and read to each participant before obtaining her signed or thumb-print consent. Confidentiality of the information provided was maintained throughout the study.

### Statistical Analysis

The outcome variable of interest was birth spacing, being the first birth interval after marriage for primiparous women, or the preceding interbirth interval for multiparous women. The former interval was calculated by subtracting the marriage age from the current age, before categorizing as either short (≤12 months) or normal (>12 months). The latter interval was computed using the age of the youngest child and the current maternal age, and then classified as either short (≤24 months) or normal (>24 months). The recommended interval between successive births is at least 24 months, since shorter intervals are known to have negative maternal and perinatal outcomes (15).

Comparisons of characteristics were made between short and normal birth spacing groups using descriptive statistics, *t*, and chi-square tests. Logistic regression analyses were then performed to ascertain factors associated with the prevalence of short birth spacing. All statistical analyses were undertaken in the STATA package Version 13.

## Results

### Sample Characteristics

About half (*n* = 363, 51.8%) of the 701 participants were primiparous. Tables 1 and 2 present the demographic and obstetric characteristics of the primiparous and multiparous women, respectively. Among the primiparous group, their mean age was 21.2 (SD 2.6) years and gestational age at recruitment 28.3 (SD 5.4) weeks. For multiparous women (*n* = 338, 48.2%), they were 26.1 (SD 4.0) years and 27 (SD 5.5) weeks, respectively. The average age at marriage was about 19.5 years for all mothers.

**Table 1:** **Demographic and obstetric characteristics of primiparous pregnant women**.

Characteristic	First birth interval		Total	*P* value
	<12 months	>12 months		
*n*	141 (38.8%)	222 (61.2%)	363	
Maternal age (years)	22.39 ± 2.56	20 ± 2.38	21.18 ± 2.63	<0.001
Gestational age (weeks)	28.24 ± 5.40	28.33 ± 5.44	28.30 ± 5.42	0.869
Paternal age (years)	27.27 ± 3.69	25 ± 3.86	25.92 ± 3.94	<0.001
Women marriage age (years)	19.51 ± 2.43	19.55 ± 2.37	19.53 ± 2.39	0.850
Caste				0.095
Upper caste	76 (53.9%)	118 (53.1%)	194 (53.5%)	
Janajati	38 (26.9%)	43 (19.4%)	81 (22.3%)	
Lower caste	27 (19.2%)	61 (17.5%)	88 (24.2%)	
Maternal education level				0.160
None/primary	27 (19.2%)	52 (23.4%)	79 (21.7%)	
Secondary	47 (33.4%)	87 (39.2%)	134 (36.9%)	
College	67 (47.4%)	83 (37.4%)	150 (41.4%)	
Paternal education level				0.663
None/primary	24 (17.0%)	44 (19.8%)	68 (18.7%)	
Secondary	49 (34.8%)	81 (36.5%)	130 (35.8%)	
College	68 (48.2%)	97 (43.7%)	165 (45.5%)	
Wealth quintile^a^				0.963
1	32 (22.7%)	46 (20.7%)	78 (21.5%)	
2	22 (15.6%)	41 (18.6%)	63 (17.4%)	
3	26 (18.4%)	41 (18.6%)	67 (18.5%)	
4	28 (19.9%)	43 (19.5%)	71 (19.6%)	
5	33 (23.4%)	50 (22.6%)	83 (22.9%)	
Residential location				0.411
Urban	78 (55.3%)	113 (50.9%)	191 (52.6%)	
Rural	63 (44.7%)	109 (49.1%)	172 (47.4%)	
Knowledge of family planning methods				0.005
No	31 (22.0%)	80 (36.0%)	111 (30.9%)	
Yes	110 (78.0%)	142 (64.0%)	252 (69.1%)	
Sex of newborn^a^				0.218
Male	72 (55.4%)	125 (62.2%)	197 (59.5%)	
Female	58 (44.6%)	76 (37.8%)	134 (40.5%)	

*^a^Missing data present*.

Of the 363 primiparous women, about half came from upper caste and urban area, with the majority obtained secondary or above education (78%) and knew about family planning methods (69%). Among the multiparous group, information on birth interval was missing for 10 cases. Approximately half of the group was from upper caste and urban area. Most of them had only one previous child (60%) and knew about family planning methods (82%). It is interesting to note that 60% of the newborns in both groups were male.

**Table 2:** **Demographic and obstetric characteristics of multiparous pregnant women**.

Characteristic	Preceding interbirth interval		Total^b^	*P* value
	<24 months	>24 months		
*n*	76 (23.2%)	252 (76.8%)	328	
Maternal age (years)	25.03 ± 4.69	26.52 ± 3.67	26.1 ± 3.97	0.004
Gestational age (weeks)	28.68 ± 5.57	27.19 ± 5.57	27.0 ± 5.54	
Paternal age (years)	29.81 ± 5.93	31.13 ± 5.03	30.83 ± 5.28	0.055
Women marriage age (years)	19.28 ± 2.85	19.16 ± 2.53	19.19 ± 2.60	0.707
Caste				0.029
Upper caste	34 (44.7%)	133 (52.8%)	167 (50.9%)	
Janajati	13 (17.1%)	61 (24.2%)	74 (22.6%)	
Lower caste	29 (38.2%)	58 (23.0%)	87 (26.5%)	
Maternal education level				0.999
None/primary	30 (39.5%)	100 (39.7%)	130 (39.6%)	
Secondary	28 (36.8%)	92 (36.5%)	120 (36.6%)	
College	18 (23.7%)	60 (23.8%)	78 (23.8%)	
Paternal education level				0.190
None/primary	25 (32.9%)	57 (22.6%)	82 (25.0%)	
Secondary	29 (38.2%)	108 (42.9%)	137 (41.8%)	
College	22 (28.9%)	87 (34.5%)	109 (33.2%)	
Wealth quintile^a^				0.738
1	15 (20.3%)	49 (19.6%)	64 (19.8%)	
2	19 (25.7%)	54 (21.6%)	73 (22.5%)	
3	16 (21.6%)	49 (19.6%)	65 (20.1%)	
4	10 (13.5%)	51 (20.4%)	61 (18.8%)	
5	14 (18.9%)	47 (18.8%)	61 (18.8%)	
Residential location				0.164
Urban	47 (61.8%)	133 (52.8%)	180 (54.9%)	
Rural	29 (38.2%)	119 (47.2%)	148 (45.1%)	
Knowledge of family planning methods^a^				0.049
No	19 (25.0%)	38 (15.2%)	57 (17.5%)	
Yes	57 (75.0%)	212 (84.8%)	269 (82.5%)	
Sex of newborn^a^				0.080
Male	33 (50.0%)	145 (62.0%)	178 (59.4%)	
Female	33 (50.0%)	89 (38.0%)	122 (40.6%)	

*^a^Missing data present*.

*^b^Missing birth spacing for 10 women*.

### Birth Spacing

For primiparous women, the mean first birth interval was 19.7 (SD 16.5) months, and 141 (38.8%) of them gave their first child birth within 1 year of marriage. The mean preceding interbirth interval for multiparous women was 44.9 (SD 21.8) months with median 42 months. Such birth spacing ranged from 6 to 98 months, with 23.2% being <24 months. The short and normal birth spacing subgroups for both primiparous and multiparous women appeared to be different in terms of caste, knowledge of family planning methods, maternal age, and paternal age (see Tables 1 and 2). Short first birth intervals seemed more common among upper caste and Janajati (74/194 and 38/81, respectively) and among women who had knowledge of family planning methods (110/252) (Table 1). Short interbirth intervals were more commonly in younger, in lower caste (29/87), and among women without knowledge of family planning methods (19/57) (Table 2).

Logistic regression analyses confirmed that maternal age was the only factor significantly associated with first birth interval among primiparous women, whereas maternal age, caste, and sex of newborn were significant factors affecting the preceding interbirth interval in the multiparous group (Table 3). In general, the prevalence of short birth spacing tended to be inversely associated with advancing maternal age. For the multiparous group, Janajati and lower caste women were more likely to incur short birth spacing than upper caste women. Those mothers with a female newborn were almost twice the risk of incurring short birth spacing than their counterparts who gave birth to a male child.

**Table 3:** **Characteristics associated with short preceding interbirth interval (<24 months), *n* = 328**.

Characteristic	Adjusted odds ratio^a^ (95% confidence interval)	*P* value
Maternal age (years)	0.91 (0.84, 0.98)	0.018
Caste		0.006
Upper caste	1	
Janajati	1.17 (0.41, 2.83)	
Lower caste	2.28 (1.26, 4.14)	
Sex of newborn		0.04
Male	1	
Female	1.80 (1.01, 3.18)	

*^a^From backward stepwise logistic regression, variables excluded were paternal age, maternal education level, paternal education level, wealth quintile, residential location, and knowledge of family planning methods*.

## Discussion

Birth intervals vary from one population to another. The median time of preceding interbirth interval in this population (42 months) was greater than the average of Nepal (36.2 months) (10) and those reported in Ethiopia (31–33 months) (16) and Malaysia (26 months) (17). Nevertheless, about one-quarter of the birth intervals were <24 months, higher than the prevalence of 16–18% observed from a randomized controlled trial of birth outcomes in the Makawanpur district of Nepal (18) and slightly <28% estimate based on the Nepal Demographic and Health Survey 2011. Short interbirth interval was found to be associated with maternal age, caste, and sex of newborn. The inverse association between age and shorter birth spacing was consistent with findings from Ethiopian (16) and Malaysian (17).

Caste was another factor affecting the preceding interbirth interval. Lower caste women are typically characterized by less education and wealth (19). Moreover, lower caste family tends to have large family size, apparently due to a lack of usage or non-awareness of family planning methods. The higher fertility, coupled with lower contraceptive use among poor and marginalized segments of the society, may be attributed to negligence to control family size or deprivation from family planning methods (20). Janajati mothers consist of mainly Tibeto-Burman women who have different customs and culture from other castes. In Nepal, total fertility rate varies between caste and ethnic groups and is highest among Muslims followed by lower caste (9).

Nepalese women who gave birth to a female baby appeared to have a shorter birth interval, similar to Ethiopian women (21), while the ratio of male newborns was higher in this population. Interestingly, knowledge of family planning methods and maternal education might be masked by other factors influencing the birth interval. In fact, the proper use of family planning methods is crucial to space births. On the other hand, maternal knowledge and attitude toward family planning methods were not significantly associated with short birth spacing in Malaysia as well (17).

For primiparous women, their first birth interval after marriage was significantly associated with age only. This finding supports that variables affecting conception are parity dependent (22). Older women tended to wait less after marriage to produce their first baby. Overall, almost 40% of the primiparous women gave birth within 1 year after marriage, suggesting their desire for a child irrespective of demographic status and obstetric factors. It is consistent with the cultural norm to meet expectation of family members, as well as providing a social identity and acceptance into the husband’s household (23).

Several limitations should be considered. In this study, birth spacing for multiparous women was defined in terms of preceding interbirth interval rather than interpregnancy interval, the latter being a sensitive issue and difficult to obtain accurate information since it must account for incidences of abortion. Furthermore, successive birth intervals were calculated based on recall of the age of their youngest child, which might incur recall and reporting biases. Another limitation concerns the lack of information on other confounding factors, such as the use of family planning methods, breastfeeding status, maternal physiological status, gender, and survival status of the previous child.

## Conclusion

Almost 40% of primiparous pregnant women gave their first child birth within 1 year of marriage, while the birth spacing among multiparous women was relatively longer with a median of 42 months. Most early births (≤12 months of marriage) tend to occur in older, upper caste women who have knowledge of family planning methods. The preceding interbirth interval was associated with maternal age, caste, and sex of the newborn infant. Optimal birth spacing programs should target lower caste and Janajati mothers, along with promotion of gender equality in society.

## Author Contributions

RK conceived and managed the project and data collection, performed statistical analysis, and drafted the manuscript. AL contributed to data analysis and revision of the manuscript. Both authors read and approved the final version for publication.

## Conflict of Interest Statement

The authors declare that the research was conducted in the absence of any commercial or financial relationships that could be construed as a potential conflict of interest. The reviewer JC and handling editor declared their shared affiliation, and the handling editor states that the process nevertheless met the standards of a fair and objective review.
